# Epithelioma of the Prepuce, and a Singular Anatomical Vagary

**Published:** 1875-09

**Authors:** A. L. Wright

**Affiliations:** Carroll, Iowa


### EPITHELIOMA OF THE PREPUCE, AND A SINGULAR ANATOMICAL VAGARY.

By A. L. WRIGHT, M.D., Carroll, Iowa.

Dec. 25. Thomas Wilson; married; age, 28; occupation, shoemaker; nativity, Scotch-Irish. Has been complaining for some months, of progressive weakness, or "laziness," as he expresses it, and vomiting on rising in the morning. The matter vomited consists of a colorless fluid, evidently water and mucus; does not feel sick or nauseated before vomiting, nor does he experience pain. A drink of water before rising would prevent the vomiting. Appetite is very good, food occasions no distress. There was great thirst, especially at night; this had existed for years, ever since he was a child.

Has always passed large quantities of urine; on examination I found it of a pale straw color, a very slight deposit on cooling, absence of glucose and albumen—(did not examine the urine as I desired, for want of chemicals and apparatus). Complains of a slight itching pain at the meatus urinarius, on micturition, has to get up several times during the night to urinate. There was a muco-purulent discharge from the urethra, very fetid; it did not pass while urinating, but during the interval. Skin dry and rough; bowels have been regular; light hurts his eyes, "they are weak," as he expresses it; conjunctiva pale; there was a dark, semi-solid substance adhering to his teeth, it would collect in a short time after cleaning them, it seemed to exude from his gums; epistaxis occurs on the least exertion; tongue clean, large and flabby. He presented that peculiar cachectic appearance so characteristic of malignancy; pulse 70; full and regular; respirations, 20. Had been troubled for some time with cramps of the flexor muscles of his legs, especially at night, occasioning great distress and loss of sleep.

The treatment was calculated to build up and give tone to the system:—Fresh air, baths, a nutritious diet, bismuth sub. nit. to allay irritability of the stomach, with ℞. Tinc. Ferri Mur., ℥j; Quiniæ Sulphas., ʒj; Strychniæ Sulphas., gr.j; Syrup Aurantii Cort., ℥iij. M. S. Teaspoonful three times a day.

Dec. 31. Discharge from penis has entirely stopped; thirst not so great; cramps do not trouble him much; rests well at night.

Jan. 4. Says something broke in his penis during the night, bled freely for a few minutes; feels better this A. M. An examination reveals that the discharge comes from between the glans penis and prepuce; in attempting to retract the prepuce, which is swollen and agglutinated to the glans by a thick, pultaceous substance, he experiences great pain and faintness, so that I am compelled to desist. Direct that he syringe the part, by passing the nozzle of a syringe between the glans and foreskin several times before my next visit.

Jan. 7th. Vomited several times last night; is very much prostrated this A. M.; am able to retract prepuce; find the glans covered with the sebaceous secretion of the glandulæ tyronii; the papillæ around the corona are enlarged; on either side of the frænum preputii, I find a small tumor about the size of a bean, on a firm, hard and inflamed base, covered with a dark, foul, ulcerating surface, discharging a muco-sanguineous fluid, very offensive, bleeding freely if the ulcerated surface is touched; the prepuce has also partaken of the disease. I considered it malignant, and advised its removal as soon as the patient's condition would admit of it. Lymphatics are not involved. I cauterized the tumor to check hæmorrhage which was considerable in the aggregate, also used disinfectants.

Jan. 8th. Hæmorrhage from his gums is more copious; his breath is very offensive; vomited this morning a dark colored fluid, resembling coffee grounds; has some pain in epigastrium when vomiting; pressure does not cause pain; pulse 70, full and regular.

Jan. 11th. Continues the same.

Jan. 12th. Patient is very much prostrated; hæmorrhage from gums and penis is very copious; blood does not coagulate. Applied basilicon ointment to penis, and gave the following three times a day: ℞. Tinc. Ferri Mur., Acid Sulph. Aromat., *aa* fl. ʒ vj; Syrup. Limonis, fl. ℥ iij. M.

Jan. 13. The under surface of the prepuce is very much swollen, great difficulty in drawing it over the glans. Appetite continues good, although he frequently vomits up everything he eats; bowels regular; rests good at night. Milk punch to be given freely.

Jan. 14th. Hæmorrhagic tendency increasing; slept none last night; had considerable pain on left side, below and left of nipple; percussion resonance normal; discovered friction sound with inspiration on that side; pulse, 70; respirations, 20; has vomited several times the dark coffee ground fluid, very offensive. Gave Morphiæ Sulph., gr. ¼, every hour, until relieved. Sp. Vini. Gallici, ℥ ss, every hour.

Jan. 16th. Is unable to void his urine; passed catheter, and drew off three pints of high colored, very offensive urine; bowels have not moved for several days. Gave an enema. Is entirely free from pain. Did not examine his chest.

Jan. 20th. Patient is failing rapidly. Died at three P. M.

Autopsy, nineteen hours after death, revealed the following curious and rare anatomical anomaly: Lungs and pleura healthy; the pericardium contained half a pint of serum; there was a copious fibrinous exudate into the pericardial sac; the heart was enlarged, the walls friable; dangling in each auriculo-ventricular opening was a white fibrinous clot about two inches in length; there was a fibrinous exudation adherent to the endocardium of each ventricle, none on the valves; there was a small earthy concretion on one segment of the mitral valve; the endocardium of the auricles was healthy in appearance.

The cardiac end of the stomach presented a dark mottled appearance, that of passive congestion ; the parietes of the same were thickened ; the pyloric end was normal. The walls of the duodenum were thickened somewhat; jejunum and ileum normal ; ascending colon was filled with scybala ; liver, spleen and pancreas presented a normal appearance ; there was cystitis. Finding no kidneys in the lumbar region, I began at the bladder, thought I would trace the ureters to their termination ; there was but one ureter : that entering directly into the bladder at the base, and not passing between the mucous and muscular coats before entering. It was about four inches in length, and about one-fourth of an inch in diameter ; following it I found but one kidney, situated back of the rectum, and just below the promontory of the sacrum, in the mesial line. The kidney was lobular, less firm than normal, weighing but three ounces. The vessels and ureter entered on its anterior surface ; there was one artery and one vein.

The pathology appended has kindly been furnished by Dr. I. N. Danforth, to whom I sent the kidney.

---

NOTE BY DR. DANFORTH.

The specimen alluded to, came safely to hand. It is manifestly more interesting from a physiological than a strictly pathological stand-point. The kidney is distinctly lobulated, like the kidneys of the fœtus, and of the lower mammalia ; six very distinct lobules are very easily made out. The organ is triangular in shape, and at the apex of the triangle it is quite thin. Its pelvis is immoderately large in proportion to the size of the organ, and the same remark is true of the size of the ureter and calyces. There is no "hilus," but the ureter and blood vessels find entrance and exit upon what I take to be the anterior aspect of the organ. Upon examining the artery and vein I find that they also are out of all proportion to the size of the organ, so that this little kidney must have received nearly as much blood as both kidneys ordinarily receive. Seeing that its weight is only two ounces and a half, (in place of four and a half ounces, the average weight of the normal kidney), it is interesting to inquire why so large an amount of blood was required, and how so large an amount of urine was secreted.*

Upon laying the kidney freely open, I find that the usual abrupt division into "cortical" and "medullary" portions is wanting; there are no "pyramids" at all; the pelvis, instead of being a single well defined cavity, spreads out into several deep "cornua" or excavations, which penetrate in different directions. These channels are simply so many branches of the ureter, which together constitute the capacious pelvis, and then branch off in different directions to gather the urine directly from the secreting portion; that is, without the usual intermediary conducting apparatus—the so-called "pyramids," which are made up mainly of the "tubuli recti," whose function is simply to conduct the urine from the convoluted tubes to the cavity known as the "pelvis."

The revelations of the microscope concerning this singular kidney are very interesting. I made thin sections from all parts of the organ and have studied many of them, in different media, (glycerine, damar, and balsam), some of them stained with logwood, others with carmine, and still others with a weak aqueous solution of iodine. I notice the following points:

*First.* The malpighian bodies are unusually large—indeed, the largest I ever saw—and the coil of blood-vessels within them unusually complicated and dense. The afferent vessels are exceptionally large, and their longitudinal and circular fibres can be demonstrated with great perfection.

* In relation to the weight given above, I should remark that considerable allowance must be made for loss of water occasioned by immersing the specimen in alcohol for preservation; the action of alcohol being to rob the tissues of water. After soaking the kidney in water for five hours, I find that it has gained three-quarters of an ounce. I presume that three and a half ounces would be about an "honest" estimate.

*Secondly.* The arteries and veins ramifying in the substance of the organ are everywhere very large as compared with the size of the organ, and their structure is very easily demonstrable.

*Thirdly.* The tubuli uriniferi are everywhere quite large, and they are coiled in the most compact and complicated manner ; indeed, they seem to be packed away with the view of storing the greatest amount of "working" material in the smallest possible space.

*Fourthly.* The endothelium of the convoluted tubes just alluded to is very distinct and prominent. This is especially true of a freshly cut section, dipped in dilute acetic acid, stained with a weak solution of iodine and then mounted in glycerine and examined *at once.* I have never seen the secreting structure of the human adult kidney so handsomely displayed before.

*Lastly.* I do not find in any single section, any of the "tubuli recti" which compose the "pyramids" of the typical kidney: the convoluted tubes seem to have poured their secretion directly into the elongated "calyces," or branches of the ureter, before spoken of, which extend into the kidney structure and supply the place of the "pyramids."

From this account it will be seen that this kidney was almost wholly composed of secreting structure and malpighian bodies. Into the smallest space the largest possible amount of secreting surface was packed. Hence the large blood vessels and the large ureter were matters of necessity. I do not think it an extravagant statement to say, that this one small kidney contained as much secreting surface as both kidneys ordinarily contain ; for it must be remembered that in the typical kidney the cortical portion forms but a comparatively small part of the organ, while in the specimen now under consideration the structure was entirely of that nature. Hence it is not difficult to believe that this kidney, by "working hard" was able to perform the duties of two kidneys in a manner acceptable to the economy.

